# Factors Associated with Willingness to Participate in Clinical Trials in Poles: A Cross-Sectional Prospective Study

**DOI:** 10.3390/jcm15072578

**Published:** 2026-03-27

**Authors:** Natalia Cięszczyk, Marcin Czech, Łukasz Pronicki, Mariusz Gujski

**Affiliations:** 1 Department of Public Health, Medical University of Warsaw, 02-091 Warsaw, Poland; 2 Institute of Mother and Child, 01-211 Warsaw, Poland

**Keywords:** clinical trials, recruitment to clinical trials, clinical studies, patient participation, motivation to participate

## Abstract

**Background:** Difficulties in recruiting patients for clinical trials increase costs and delay the implementation of new therapies. A better understanding of participants’ motivations and barriers can help with developing effective recruitment strategies. The aim of the study was to identify the factors influencing the decisions of adult Poles to participate in clinical trials. **Methods:** The survey was conducted among Polish adults aged 18 years and older by the independent research company Ariadna between January and February 2023. The questionnaire consisted of 22 questions, nine of which related to the determinants of participation in clinical trials. 1079 people took part in the survey. **Results:** The study population included 568 women (52.6%) and 511 men (47.4%). The mean age of respondents was 44.96 years (SD = 16.30). 49.9% of respondents (*n* = 538) declared their willingness to participate in clinical trials in the future. Among those who were reluctant (*n* = 158, 14.6%), the main barriers were: safety concerns (*n* = 59, 5.5%), lack of trust (*n* = 43, 4.0%), and insufficient knowledge (*n* = 33, 3.1%). The strongest motivation was the desire to improve health (*n* = 869, 80.5%), and the most frequently indicated reason for participation was cancer (*n* = 740, 68.6%). The least frequently indicated were diseases of the urinary and reproductive systems (*n* = 125; 11.6%). **Conclusions**: The results highlight key aspects important to patients when deciding whether to participate in clinical trials. Such findings may prove useful for researchers in getting to know their patients better and in developing effective strategies to recruit and retain participants in clinical trials.

## 1. Introduction

In recent years, there has been a rapid development of medicine, which is largely supported by clinical trials. Clinical trials make it possible to assess the efficacy and safety of new drugs, therapies, and medical procedures [1]. Poland has become one of the most active countries conducting commercial clinical trials by the pharmaceutical industry as part of the globalization trend. In 2019, Poland ranked 11th worldwide in terms of its share in the global market of industry-sponsored commercial clinical trials. The leading countries in this ranking were the United States, Germany, and Japan. Within the European context, Poland is regarded as a dynamically developing clinical research environment. Moreover, between 2014 and 2019, Poland recorded one of the highest increases in its share of the global commercial clinical trials market, ranking fifth globally, following China, Spain, South Korea, and Taiwan. In Europe, the largest clinical trial markets are mainly found in Western European countries such as Germany, France, and the United Kingdom; however, in recent years, the importance of Central and Eastern European countries, including Poland and neighboring countries such as the Czech Republic and Hungary, has been growing [2]. In November 2024, the ClinicalTrials.gov website listed more than 517,000 ongoing clinical trials worldwide [3]. In Poland, in 2023, there was a record-breaking number of clinical trial applications registered by the President of the Office for Registration of Medicinal Products, Medical Devices, and Biocidal Products, which amounted to 784 [4]. This compares with 597 applications to start a clinical trial for a medicinal product in 2020 [5] and 478 applications to start a clinical trial for a medicinal product in 2017 [6]. Clinical trials depend on the participation of potential trial participants. Recruiting patients for clinical trials has been a challenge for sponsors and researchers for years. This phase consumes as much as 30% of the time of the entire trial and uses up about 40% of resources allocated to the development of a substance, averaging $1.2 billion [7]. In 2019, the costs associated with attracting research participants reached approximately $52bn [2]. According to the McKinsey report, the low efficiency of patient recruitment is one of the biggest problems in the clinical research industry. Between 2019 and 2022, the number of required participants increased from 2.2 million to 2.6 million, posing additional challenges for trial sites. Recruitment time increased from an average of 12 to 18 months, which slows down innovation and leads to incomplete trials [8]. To better understand the scale of the problem, it is useful to refer to literature data from recent years on recruitment efficiency. Currently, around 11% of all clinical trials initiated face the problem of failing to engage even one participant. Nearly 90% of trials experience delays due to ineffective recruitment. Most ongoing trials never reach the planned number of participants, and several percent are terminated prematurely for this reason. As a result, recruitment difficulties account for about 75% of study drop-outs [9]. Recruitment of participants directly affects the success of a research project. Low participation rates can result in selection bias, which in turn complicates the proper analysis of data obtained from samples that do not reflect the standard population. In addition, a limited number of participants undermines the accuracy of the results and reduces the statistical power of the analyses [10]. However, recruitment challenges in clinical trials are multifactorial and may result not only from patients’ willingness to participate but also from aspects related to study design, eligibility criteria, and organizational factors of trial conduct. Nevertheless, perceptions and attitudes of potential participants towards clinical trials remain an important component of this complex process and play a key role in the effectiveness of recruitment strategies and in maintaining participant engagement throughout the trial [11].

Knowledge about Poles’ attitudes towards participation in clinical trials is limited, even though they are potential participants in future research on treatment and prevention. Understanding motivations and barriers is crucial for effective communication about the importance of research and increasing public participation. The aim of this study was to identify the reasons why Poles decide to participate or refuse to participate in clinical trials.

## 2. Materials and Methods

### 2.1. Study Design

The survey was conducted on a nationwide sample of Poles. We used the method of computer-assisted web interviewing (CAWI), which enabled us to effectively reach a wide range of participants and collect data efficiently. For the questionnaire we prepared, we used the services of the Ariadna research panel—an independent research platform with nationwide coverage. The Ariadna panel allows research to be conducted with the utmost care for the accuracy and reliability of results. When conducting the research, the Ariadna panel had a valid Interviewer Quality Control Program certificate, which confirms the high quality of the research services provided. This certificate is awarded based on annual, independent audits conducted by the Polish Association of Public Opinion and Marketing Researchers [12]. Survey respondents received personalized survey links to their email addresses, allowing them to confirm their identity before taking the survey. Once completed, Ariadna provides the data in the form of anonymized statistical summaries. These measures comply with the Data Protection Act and the ICC/ESOMAR Code of Ethics, developed by the European Association of Public Opinion and Market Researchers and the International Chamber of Commerce in Poland [13]. A statistical analysis was then carried out.

### 2.2. Ethical Aspects

The study commenced after receiving approval from the Bioethics Committee. On 16 January 2023, by decision number AKBE/2/2023, the research project, together with the questionnaire, received approval from the Ethics Committee of the Warsaw Medical University. The data collected as part of the survey were fully anonymous, making it impossible to identify individual study participants. All procedures related to the study, involving the participation of the participants, were carried out in accordance with the ethical standards set by the relevant institutions or national research regulators and in accordance with the principles of the Declaration of Helsinki.

### 2.3. Participants

The survey was conducted between January and February 2023 on a nationwide sample of 1079 Polish adults aged 18 years and older. To ensure representativeness, the demographic structure of the sample, including gender, age, and place of residence, was adjusted to reflect the characteristics of the adult population of Poland. The inclusion criteria were age ≥ 18 years and residence in Poland. Participation in the survey was voluntary and anonymous, and completion of the questionnaire was considered as providing informed consent to participate in the study. Incomplete questionnaires were excluded from the analysis.

### 2.4. Questionnaire Development

At the outset, the research objectives were defined, and the necessary information to meet them was identified to be included in the questionnaire. The research tool was a nationwide self-completion questionnaire created specifically for this study. The questionnaire was administered in Polish. During the development of the questionnaire, an analysis of previous studies on determinants of willingness to participate in clinical trials in different communities was conducted. The content of the questionnaire was based on questions from publicly available studies of factors associated with willingness to participate in clinical trials conducted in the United States [14,15] and South Korea [16]. The questions were adapted to Polish conditions through an analysis of their linguistic and cultural adequacy and consultations with experts experienced in clinical trials, public health, and survey methodology. At this stage, the comprehensibility of the questions, their substantive accuracy, and their consistency with the purpose of the study were evaluated as part of the content validity assessment. The questions were logically structured to make the transition from general issues to specific ones smooth. The questionnaire consisted of 22 questions, including nine factors related to willingness to participate in clinical trials. Some of the questions also collected demographic information such as gender, age, size of residence, education level, province, and marital status. Before commencing the actual study, a pilot questionnaire was conducted among a small group of individuals (*n* = 43) not professionally involved in medicine. The aim of the pilot study was to assess the clarity of the questions and the time needed to complete the questionnaire and to identify potential interpretation difficulties. Based on the comments received, minor editorial corrections were made to improve the clarity of the wording. The data from the pilot study were not included in the main analysis. This pilot procedure served as a preliminary validation step to ensure the clarity and appropriateness of the questionnaire items. Willingness to participate in clinical trials and factors that might influence this decision were assessed through seven questions with a five-point response scale ranging from ‘Definitely yes’ to ‘Hard to say’. The remaining two questions addressed the identification of barriers to participation in clinical trials and the circumstances under which the respondent would take part in clinical trials.

### 2.5. Statistical Analysis

To assess the psychometric properties of the set of questions concerning motivation to participate in clinical trials, the reliability of the scale was evaluated. Internal consistency was assessed using Cronbach’s alpha coefficient. The analysis included six items related to potential motivators for participation in clinical trials (improvement of one’s own health, access to innovative therapy, helping future patients, encouragement from loved ones, a physician’s recommendation, and contribution to the development of medical knowledge). Construct validity was assessed using exploratory factor analysis to verify whether the analyzed items measured a common, unidimensional construct of motivation to participate in clinical trials. The question regarding the declared willingness to participate in a clinical trial in the future was analyzed as a separate outcome variable and was not included in the scale reliability analysis. Continuous variables were summarized using mean and standard deviation. Categorical variables were presented as frequencies and percentages. Correlations between declared willingness to participate in clinical trials and perceived motivations for participation were analyzed using Spearman’s rank correlation coefficient (ρ). Statistical significance was set at *p* < 0.05. To account for multiple testing arising from the analysis of several motivational factors in relation to the outcome variable, *p*-values were adjusted using the Benjamini–Hochberg procedure. All statistical analyses were performed using R software (version 4.3.2). In the case of conditional questions (asked only to a selected group of respondents), individuals to whom the question did not apply were treated as ‘not applicable’ and were not considered missing data.

## 3. Results

The analyzed set of six items showed high internal consistency (Cronbach’s alpha = 0.87), and exploratory analysis confirmed the unidimensional structure of the tool. A total of 7330 invitations to complete the survey were sent out. One thousand and seventy-nine (1079) respondents took part in the survey, including 568 women (52.6%) and 511 men (47.4%). The response rate was 26.04%. The mean age of respondents was 44.96 years (SD = 16.30), with no age differences between men and women. Of the 1079 respondents, 404 (37.4%) lived in rural areas, 134 (12.4%) in cities with more than 500,000 inhabitants, and 140 (13.0%) in cities with up to 20,000 inhabitants. There were 96 people (8.9%) living in medium-sized cities with a population of 20,000–49,000, while 87 respondents (8.1%) lived in cities with a population of 100,000–200,000. The largest group, 155 people (14.4%), were residents of the Mazowieckie Voivodeship. The fewest, only 24 people (2.2%), represented the Opolskie Voivodeship. In terms of education, 383 people (35.5%) had a university degree, and 338 (31.3%) had a secondary education. Post-secondary education was declared by 111 people (10.3%), and the same number of respondents (10.3%) had a basic education. There were 96 (8.9%) bachelor’s degree graduates, and 40 people (3.7%) had primary or lower secondary education. In terms of marital status, most respondents (*n* = 575, 53.3%) were in a relationship. Unmarried people accounted for 30.9% (*n* = 333), divorced people accounted for 10.8% (*n* = 116), and widows and widowers accounted for 5.1% (*n* = 55). Detailed characteristics of the participants are shown in Table 1.

### Willingness to Participate in Clinical Trials

The questionnaire included the question, ‘Would you choose to participate in clinical trials in the future?’ Less than half of the participants (*n* = 538, 49.9%) declared a willingness to participate (responses ‘Rather yes’ or ‘Definitely yes’). In contrast, 11.0% (*n* = 119) reported that they would probably not agree, and 3.6% (*n* = 39) stated that they would definitely not participate. More than one-third of respondents (*n* = 383, 35.5%) were undecided.

Participants who indicated that they would definitely not (*n* = 39, 3.6%) or probably not (*n* = 119, 11.0%) agree to participate in clinical trials were asked to specify the main reason for their decision. The most frequently reported concern was related to personal safety (*n* = 59, 5.5%). Other reasons included lack of trust in clinical trials (*n* = 43, 4.0%) and insufficient knowledge about clinical research (*n* = 33, 3.0%) (Table 2).

Respondents rated various factors that could influence their decision to participate in clinical trials (Table 3). The first question was ‘Would the desire to improve your health be a motivating factor?’ The vast majority (*n* = 869, 80.5%) said yes (‘Rather yes’: *n* = 492, 45.6%; or ‘Definitely yes’: *n* = 377, 34.9%). A small group of respondents (*n* = 62, 5.7%) felt that this would not be a motivating factor for them. The remaining respondents (*n* = 148, 13.7%) were undecided and indicated that it was difficult for them to answer this question.

The second question was ‘Would the possibility of receiving an innovative therapy be a motivating factor?’ Most respondents (*n* = 788, 73.0%) agreed (‘Rather yes’: *n* = 529, 49.0%, or ‘Definitely yes’: *n* = 259, 24.0%), emphasizing that the possibility of receiving an innovative therapy motivates them to participate in the study. A small proportion of participants (*n* = 88, 8.2%) disagreed (‘Rather no’: *n* = 77, 6.6%, or ‘Definitely no’: *n* = 17, 1.6%). The rest of the participants (*n* = 203, 18.8%) were undecided about the influence of this factor on their decision to participate in the study.

The third question was ‘Would helping future patients be a motivating factor?’ Almost 70% of respondents (*n* = 738, 68.4%) referred positively to the possibility of helping future patients as a motivation to participate (‘Rather yes’: *n* = 519, 48.1% or ‘Definitely yes’: *n* = 219, 20.3%).

The fourth question was ‘Would the encouragement from family and friends be a motivating factor?’ 44.5% of participants (*n* = 480 disagreed (‘Rather no’: *n* = 333, 30.9% or ‘Definitely no’: *n* = 147, 13.6%)) that encouragement from family and friends would be an important motivator for them to participate in clinical trials. 30% of respondents (*n* = 324) believed that this would be a motivating factor for them. A large proportion of participants (*n* = 275, 25.5%) were undecided and could not clearly identify the influence of this factor on their decision to participate. With this factor, the largest group of people was undecided.

The fifth question was ‘Would a doctor’s recommendation be a motivating factor?’ Most participants (*n* = 667, 61.8%) agreed (‘Rather yes: *n* = 499, 46.2%’ or ‘Definitely yes: *n* = 168, 15.6%’) that a doctor’s recommendation was an important motivating factor for participation.

The sixth and final question on motivating factors was ‘Would the desire to contribute to medical knowledge be a motivating factor?’ Most participants (*n* = 609, 56.4%) agreed (‘Rather yes: *n* = 450, 41.7%’ or ‘Definitely yes: *n* = 159, 14.7%’) that the desire to contribute to the advancement of medicine motivates them to participate in research.

From the data obtained, the desire to improve one’s own health (*n* = 869, 80.5%) is the most important motivating factor for taking part in clinical trials. In the second place is the possibility of receiving innovative therapy (*n* = 788; 73.0%), followed by the importance of helping future patients (*n* = 738, 68.4%). Next in the ranking are the factors concerning physicians’ recommendations (*n* = 667, 61.8%) and the desire to contribute to the development of medical knowledge (*n* = 609, 56.4%). The least important factor was encouragement from family and friends (*n* = 324, 30.0%). An analysis of Spearman’s rank correlation between the declared willingness to participate in clinical trials and the analyzed motivational factors was performed (Table 4).

The final question in the questionnaire was ‘In what circumstances would you take part in a clinical trial?’ Each respondent rated each circumstance in terms of ‘yes’ or ‘no’ (Table 5). Most would take part in clinical trials because of their cancer (*n* = 740, 68.6%), neurological disease (*n* = 470, 43.0%), and if they received monetary compensation (*n* = 420, 38.9%) and cardio-vascular disease (n = 420, 38.9%). The least significant reasons for taking part in clinical trials were urinary and reproductive disease (*n* = 125, 11.6%), gastroenterological disease (*n* = 132, 12.2%), and dermatological disease (*n* = 147, 13.6%).

## 4. Discussion

According to the survey, less than half of the participants (49.9%) would agree (‘Rather yes’ or ‘Definitely yes’) to participate in clinical trials in the future. In total, 14.6% of respondents believe they would not agree (‘Rather no’ or ‘Definitely no’), and a third of people (35.5%) cannot decide. Similar results were obtained in an American study, where the results of the survey show that 46% of respondents said they would be willing to participate in a clinical trial, 25% said they would not, and 29% said they were not clear on this issue [17]. Similarly, another US study from 2023 showed that 50.8% of the study population expressed a willingness to participate in future clinical trials [11]. However, higher rates of willingness to participate in clinical trials were reported in a Tunisian study (80%) [18]. It can be concluded that the differences in willingness to participate in clinical trials in different countries may be due to several cultural and socioeconomic factors, as well as differences in operating health care systems. In our study, as many as 35.5% are unable to decide whether they want to participate in clinical trials in the future. This indicates that a lack of knowledge results in uncertainty and problems in making informed and well-considered decisions regarding participation in clinical trials. It is therefore crucial to emphasize the need to educate the public about the nature and aims of clinical trials, their ethical principles, as well as the potential benefits and risks for participants and society [19]. The implementation of effective educational initiatives can increase knowledge and awareness, which in turn can increase the willingness to participate in clinical trials and improve understanding of the processes involved [20]. Those who indicated that they would definitely not (*n* = 39, 3.6%) or probably not (*n* = 119, 11.0%) agree to participate in clinical trials were asked about the main reason. Most respondents (*n* = 59, 5.5%) indicated concern for their own safety, lack of confidence in clinical trials (*n* = 43, 4%), and lack of sufficient knowledge (*n* = 33, 3.0%) as reasons. The fewest people indicated (*n* = 15, 1.4%) a lack of time and family reluctance for their participation in clinical trials (*n* = 3, 0.3%). In another study, safety concerns were also indicated as the main factor limiting participation in clinical trials [21]. In another study, participants indicated ‘risk of unknown side effects’ as the strongest barrier and time commitment as a minor barrier [22]. It seems logical that often, for patients, diagnosis and resolution of a crisis, e.g., through participation in clinical trials, takes precedence over time commitment. Interestingly, in a study conducted in Qatar, the biggest barrier to taking part in clinical trials was lack of time, cited by 47.8% of respondents, followed by ‘fear’ (13.0%), lack of knowledge about clinical trials (8.7%), and lack of interest in trials (8.7%) [23]. In the Jordanian population, on the other hand, the main reasons for not participating in research were concern about risks to one’s own health (61.1%) and lack of belief in the results and benefits of clinical trials (29.7%) [24]. The literature emphasizes that proper qualifications of participants and reliable communication of information about the study, including potential benefits and risks, are an important part of the informed consent process and can affect the safety of participants and the quality of the research conducted [25]. Our study found that the biggest motivating factor for taking part in clinical trials is the desire to improve one’s health (*n* = 869, 80.5%), the opportunity to receive an innovative therapy (*n* = 788; 73.0%), and the desire to help future patients (*n* = 738, 68.4%). Another study identified three main motivating factors for clinical trial participation that overlapped with ours, namely: personal benefit, contribution to innovation, and altruism [21]. Similar findings were also observed in studies conducted in several European countries, including Belgium, France, the Netherlands, Germany, and Hungary, where one of the most frequently cited motivations for participating in clinical trials was altruism, linked to a desire to help other patients [26]. In another study, several patients were also driven by altruistic motivations. However, most patients are primarily interested in finding the best possible treatment for their disease [27]. According to our study, the next most important motivating factors for participation in clinical trials are the physician’s recommendation factor (*n* = 667, 61.8%) and the desire to contribute to the development of medical knowledge (*n* = 609, 56.4%). Encouragement from family and friends was found to be the least important factor (*n* = 324, 30.0%). In another study where motivating factors for participating in clinical trials were examined, the weakest reason appeared to be encouragement from family (12.4%), as well as the doctor (14.2%) who would like the person to participate. The strongest reason against participation was ‘fear of side effects’ (52.6%) [11]. In another U.S. study from 2023, where respondents were asked, ‘Which of the following would motivate you to participate in a clinical trial?’ 23.4% would take a doctor’s recommendation, 22.1% of respondents would want to advance research through it, and 6.1% of respondents would take a family recommendation [28]. The analysis shows that among the various health conditions, cancer is the biggest motivator to participate in clinical trials (*n* = 740, 68.6%). In contrast, conditions considered less serious or chronic, such as gastroenterological (*n* = 132, 12.2%) or genitourinary (*n* = 125, 11.6%) diseases, appear to be less motivating for patients to participate in trials. The present analysis indicates a variation in motivational perceptions among patients, depending on the type of disease experienced. A clear trend emerges whereby those affected by diseases of high severity, such as neoplasms, show a greater willingness to participate in clinical trials, which may reflect their search for new, potentially more effective treatments. All observed correlations were statistically significant.

### Limitations

The survey was conducted using the CAWI method, which, like other online surveys, has certain limitations [29,30]. One of these is the need for internet access and computer literacy. It should be noted, however, that according to a report by the Public Opinion Research Center, 77.0% of adult residents in Poland report using the internet regularly (at least once a week) [31]. The lack of direct contact with the interviewer may also be a limitation; however, the questionnaire included a brief introduction explaining the purpose and methodology of the survey, and the inability to obtain hints assured equal conditions for all participants. Another limitation was the relatively low response rate (26.04%). In future studies, it would be worth considering sending reminders to those who did not respond to the initial invitation. It should also be noted that respondents assessed their willingness to participate in clinical trials in a hypothetical situation, as representatives of the public, rather than as patients making actual treatment decisions. In clinical practice, individuals struggling with illness may assess potential participation in a trial differently, particularly in the context of access to new treatments [32]. Furthermore, previous studies indicate that decisions regarding participation may also depend on elements of trial design, such as randomization or the possibility of being assigned to a control group [33].

## 5. Conclusions

The results of our survey highlight key aspects important in deciding whether to participate in clinical trials among adults in Poland. Understanding patient attitudes toward clinical trial participation is a key component of clinical trial design, which in turn significantly affects the effectiveness of recruitment and retention of participants during clinical trials. Adopting a patient-centered approach in conducting trials can facilitate open communication and increase understanding of trial participation. Our study presents some barriers to clinical trial participation that need to be addressed to increase participation rates, particularly safety issues, lack of trust in clinical trials, and insufficient knowledge. The results obtained allow us to identify areas that may be particularly important when planning information and recruitment strategies for clinical trials. The identified factors related to willingness to participate, such as perceived health benefits, access to innovative therapies, physician recommendation, and altruistic motivations, can serve as a reference point when developing communications targeted at potential participants. Including these elements in the information message and in the recruitment process may help to better align activities with the expectations of research teams. While further research in diverse groups, such as clinical trial participants themselves, is needed, this study represents an important first step in incorporating patient opinions into the design and recruitment of clinical trials. Future efforts, including educational activities, effective communication, and systematic improvements in public awareness, may improve willingness to participate. These findings provide valuable perspectives on factors influencing patient decisions, which are extremely important for clinical researchers. A deeper understanding of the social perspectives of those likely to participate in future clinical trials can enhance recruitment. To the authors’ knowledge, this is one of the most recent studies to analyze factors associated with willingness to participate in clinical trials among adults in Poland. There is a need for further research in diverse groups, such as clinical trial participants.

## Figures and Tables

**Table 1:** Participants’ characteristics (*n* = 1079).

		*n*	(%)
Age (years), mean SD	44.96		
18–24		141	13.1
25–34		216	20.0
35–44		180	16.7
45–54		187	17.3
≥55		355	32.9
Sex			
Female		568	52.6
Male		511	47.4
Region			
Village		404	37.4
Small town ^1^		140	13.0
Medium town lower ^2^		96	8.9
Medium town upper ^3^		115	10.7
Large city lower ^4^		87	8.1
Large city upper ^5^		103	9.5
Big city ^6^		134	12.4
Education			
Primary or middle school		40	3.7
Basic		111	10.3
Secondary school		338	31.3
Post-secondary school		111	10.3
Bachelor’s degree		96	8.9
Master’s degree		383	35.5
Voivodeship *			
Dolnośląskie		62	5.7
Kujawsko–pomorskie		59	5.5
Łódzkie		68	6.3
Lubelskie		71	6.6
Lubuskie		31	2.9
Małopolskie		97	9.0
Mazowieckie		155	14.4
Opolskie		24	2.2
Podkarpackie		63	5.8
Podlaskie		48	4.4
Pomorskie		55	5.1
Śląskie		136	12.6
Świętokrzyskie		34	3.2
Warminsko–mazurskie		30	2.8
Wielkopolskie		104	9.6
Zachodniopomorskie		42	3.9
Family status			
Single		333	30.9
Married		575	53.3
Divorced		116	10.8
Widowed		55	5.1

Population: up 20,000 inhabitants ^1^, 20,001–50,000 inhabitants ^2^, 50,001–100,000 inhabitants ^3^, 100,001–200,000 inhabitants ^4^, 200,001–500,000 inhabitants ^5^, more than 500 000 inhabitants ^6^. * Voivodeship—administrative region of Poland corresponding to the first-level territorial division of the country.

**Table 2:** Barriers to participation in clinical trials for adults in Poland (*n* = 158).

	*n*	(%)
**Barriers to participation in clinical trials**		
Concern for one’s own safety	59	5.5
Lack of time	15	1.4
Lack of confidence	43	4.0
Lack of sufficient knowledge	33	3.0
Family reluctance	3.0	0.3
Hard to say	5.0	0.5
Not applicable (respondents not asked this question)	921	85.3

**Table 3:** Factors motivating participation in clinical trials for adults in Poland (*n* = 1079).

	Definitely Yes		Rather Yes		Probably Not		Definitely Not		Hard to Say	
	*n*	(%)	*n*	(%)	*n*	(%)	*n*	%	*n*	(%)
The desire to improve one’s own health	377	34.9	492	45.6	51	4.7	11	1.0	148	13.8
Possibility of receiving innovative therapy	259	24.0	529	49.0	71	6.6	17	1.6	203	18.8
Helping future patients	219	20.3	519	48.1	76	7.1	25	2.3	240	22.2
Encouragement from family and friends	76	7.0	248	23.0	333	30.9	147	13.6	275	25.5
Physician’s recommendation	168	15.6	499	46.2	137	12.7	56	5.2	219	20.3
The desire to contribute to the development of medical knowledge	159	14.7	450	41.7	150	13.9	61	5.7	259	24.0

**Table 4:** Correlations between declared willingness to participate in clinical trials and motivational factors.

	Spearman’s ρ	Adjusted *p*-Value
**Motivational factor**		
The desire to improve one’s own health	0.396	<0.001
Possibility of receiving innovative therapy	0.428	<0.001
Helping future patients	0.461	<0.001
Encouragement from family and friends	0.383	<0.001
Physician’s recommendation	0.454	<0.001
The desire to contribute to the development of medical knowledge	0.478	<0.001

**Table 5:** List of circumstances influencing participation in clinical trials among adults in Poland (*n* = 1079).

Type of Circumstance	Yes		No	
	*n*	(%)	*n*	(%)
Monetary compensation	420	38.9	659	61.1
Cardiovascular disease	420	38.9	659	61.1
Neurological disease	470	43.6	609	56.4
Gastroenterological disease	132	12.2	947	87.8
Respiratory disease	250	23.2	829	76.8
Dermatological disease	147	13.6	932	86.4
Infectious disease	233	21.6	846	78.4
Urinary and reproductive disease	125	11.6	954	88.4
Chronic disease, e.g., diabetes mellitus	300	27.8	779	72.2
Cancer	740	68.6	339	31.4

## Data Availability

The datasets used and/or analyzed during the current study are available from the corresponding author upon reasonable request.
